# Data-Aware Retrodiction for Asynchronous Harmonic Measurement in a Cyber-Physical Energy System

**DOI:** 10.3390/s16081316

**Published:** 2016-08-18

**Authors:** Youda Liu, Xue Wang, Yanchi Liu, Sujin Cui

**Affiliations:** State Key Laboratory of Precision Measurement Technology and Instruments, Department of Precision Instrument, Tsinghua University, Beijing 100084, China; liu-yd11@mails.tsinghua.edu.cn (Y.L.); liuyanchi13@mails.tsinghua.edu.cn (Y.L.); cuisj14@mails.tsinghua.edu.cn (S.C.)

**Keywords:** out-of-sequence measurement, cyber-physical energy systems, harmonic measurement, data-aware

## Abstract

Cyber-physical energy systems provide a networked solution for safety, reliability and efficiency problems in smart grids. On the demand side, the secure and trustworthy energy supply requires real-time supervising and online power quality assessing. Harmonics measurement is necessary in power quality evaluation. However, under the large-scale distributed metering architecture, harmonic measurement faces the out-of-sequence measurement (OOSM) problem, which is the result of latencies in sensing or the communication process and brings deviations in data fusion. This paper depicts a distributed measurement network for large-scale asynchronous harmonic analysis and exploits a nonlinear autoregressive model with exogenous inputs (NARX) network to reorder the out-of-sequence measuring data. The NARX network gets the characteristics of the electrical harmonics from practical data rather than the kinematic equations. Thus, the data-aware network approximates the behavior of the practical electrical parameter with real-time data and improves the retrodiction accuracy. Theoretical analysis demonstrates that the data-aware method maintains a reasonable consumption of computing resources. Experiments on a practical testbed of a cyber-physical system are implemented, and harmonic measurement and analysis accuracy are adopted to evaluate the measuring mechanism under a distributed metering network. Results demonstrate an improvement of the harmonics analysis precision and validate the asynchronous measuring method in cyber-physical energy systems.

## 1. Introduction

Cyber-physical systems (CPS) aim at improving the system safety, security, sustainability and efficiency in large-scale in-network applications, such as smart grids, medical and health, industrial manufacturing, traffic control, smart buildings, etc. [1]. Such applications require sensing and information analysis in a wide area network and claim higher safety and quality of the measurement network. Beyond the traditional sensing network solution, cyber-physical systems combine the communication, computation and control process and offer a better performance [2]. On the demand side of the smart grid, the cyber-physical energy system (CPES) is dedicated as a special case of CPS dealing with the electrical safety and quality problems among large-scale industrial and commercial power utilization [3]. To provide the consumers with a secure and trustworthy power supply, the distributed metering architecture is exploited for large-scale, hybrid network measurement [4].

On the demand side, the automated meter reading (AMR) system, which behaves only as one-way manual centralized detection, is at the last gasp of its use corrections , while the advanced metering infrastructure (AMI) with a distributed architecture and integrated electrical information analysis service is gradually taking its place [5]. For the prospect of the electrical metering system, the smart grid is a promising solution for a less centralized and more consumer-interactive network. Within this measuring framework, electrical characteristics are collected through scattered smart meters, then meter data management systems dispose integrated computing and make deploying strategies accordingly to ensure high quality power. Harmonic measurement is an essential part of power quality assessment. Harmonics on the demand side mainly come from nonlinear appliances. Distorted harmonics introduce fluctuations, sags and disequilibrium into the grid, leading to potential damages and power failures. Fast and online harmonic analysis is significant in power grids and facing new challenges under distributed metering architectures.

**Figure 1:**
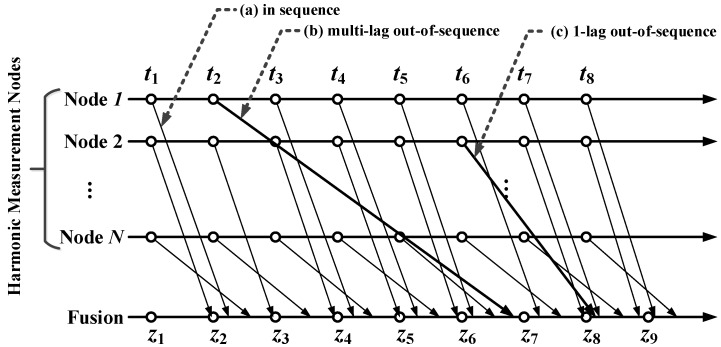
An illustration of the out-of-sequence measurement problem. (**a**) Normal sequence measurement; (**b**) multi-lag out-of-sequence measurement (three-lag delayed in this case); (**c**) one-lag out-of-sequence measurement.

The out-of-sequence measurement (OOSM) problem is one of the challenges in large-scale distributed sensor networks [6]. The AMI architecture contains thousands of electrical measuring devices. Electrical data from different sensing nodes reaches the data management center with latencies that result in temporal disorders of the data sequence, as shown in Figure 1. Data fusion, such as harmonic analysis and identification, will be influenced by disordered measurement. In target tracking sensor networks, OOSM methods have been developed and verified. OOSM has been implemented by the Kalman filter and the particle filter. Bar-Shalom [7] introduced the OOSM in multisensor systems and studied data retrodiction based on statistics theory. Bar-Shalom proposed an optimal method, A1, and suboptimal methods, B1 and C1 [8], based on the Kalman filter for one-lag disorder, then developed multi-lag OOSM Methods Al1 [9], Bl1 [10] and NBl [11]. Lanzkron proposed a two-step multi-lag OOSM method [12]. Subhash proposed an augmented state Kalman filter with a Bayesian solution [13], and Keshu improved the performance of this algorithm [14]. OOSM algorithms based on the particle filter were developed following the Kalman filter-based methods. Mallick proposed suboptimal B-PF2 based on the heuristics that the target states at different time instants are independent given the past measurements in [15]. B-PF1 was depicted in [16] by Matthew, and also, A-PF is proposed in [17,18]. Gustafsson presented a storage-efficient particle filter algorithm (SEPF) in [19,20]. Anders promoted an RBPF (Rao–Blackwellized particle filter) method in [21]. Decentralized methods for OOSM are presented in [22,23]. Handel [24] exploited the Bayesian framework to minimize the error estimation of the disordered data. Zhang proposed a complete in-sequence information (CISI) method in [25]. In the applications, Besada-Portas studied the out-of-sequence problem in mobile robot localization [26]. Klaus [6] applied OOSM for multi-sensor fusion in vehicles and proposed joint integrated probabilistic data association (JIPDA) fusion. Schueller [27] provided a temporal solution to calibrate the data sequence in an advanced driver assistance system. Liu [28] combined the OOSM algorithm with compressive sensing for harmonic measurement and identification.

Harmonic analysis requires harmonic data in the same temporal section. Out-of-sequence data will reduce the accuracy of harmonic monitoring and power quality assessment. Existing OOSM methods based on the Kalman filter or the particle filter are computationally complicated and rely on dynamic models of the tracking sources. For electrical harmonics, the dynamic model is irregular, and it is difficult to depict the precise relations of the harmonics. In addition, network-based monitoring indicates relative correlations between the signals in multiple channels. The Kalman filter- and the particle filter-based OOSM methods do not consider the network data as a whole, which is incomplete utilization of the harmonic information. The artificial neural network gains the capability to imitate a complex nonlinear system without a priori modeling, which is suitable for implementing measuring data reordering in a large-scale distributed network [29,30]. The nonlinear autoregressive model with exogenous inputs (NARX) model [31,32,33,34,35] has long been adopted in the prediction and filtering of temporal data sequences. In this paper, NARX-based OOSM methods are adopted to perform data-aware retrodiction for distributed harmonic analysis.

The main contribution of this paper lies in introducing the artificial neural network into out-of-sequence measurement in harmonic analysis. The NARX neural network is employed to predict and retrodict the disordered data without the kinematic model of the harmonic behavior. The data-aware NARX network maintains the model in the hidden layers and provides a precise approximation of the real electrical harmonic changes. The theoretical analysis on the NARX-based OOSM method’s computational complexity and memory consumption is presented. Experiments on a practical distributed electrical sensing network evaluate the performance of the proposed method.

The rest of this paper is organized as follows. Section 2 contains the OOSM problem statement and basic notations. Section 3 depicts the OOSM method and proposes the NARX-based OOSM algorithm. This section also analyzes the NARX-based algorithm’s computational complexity and compares it to the former OOSM methods. Section 4 shows the experiment results on the harmonic identification accuracy of disordered measuring data, demonstrating the validation of the measurement method proposed. Finally, Section 5 is the conclusion and the overview of future work.

## 2. Preliminaries

### 2.1. Distributed Metering Framework in a Cyber-Physical Energy System

**Figure 2:**
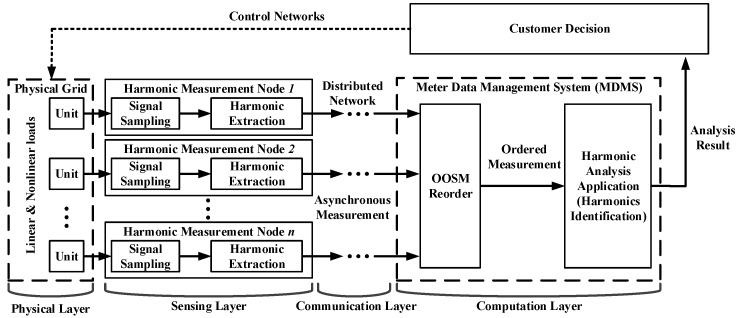
Distributed measuring schedule for harmonic analysis in the cyber-physical energy system, including sensing, communication, computation and control. The out-of-sequence measurement (OOSM) occurs in the asynchronous communication network and is rectified before the analysis process.

A large-scale sensing network for electrical information monitoring in the residential and industrial grid has attracted sufficient researchers’ focus, and the electric metering system has been evolving ever since. The conventional metering structure is the automated meter reading (AMR) system, which behaves only as one-way manual centralized detection. The advanced metering infrastructure (AMI) utilizes a distributed architecture and integrated electrical information analysis service and is gaining in popularity. In the prospect of the electrical metering system, the cyber-physical energy system is a promising solution for a less centralized and more consumer-interactive network. With the growth of the grid scale, a distributed, dynamic-configuration, demand-required response solution is offered. In the dynamic sensing network [36], the cyber system will be able to collect real-time electrical power quality parameters and to deploy distributed data analysis and computation for energy savings. In a home area network, electrical data are measured and preprocessed at the ubiquitous intelligent sensing nodes, propagating through a self-organized, multi-hop sensing network based on the IEEE 802.15.4 protocol [37] and analyzed at the data management center to assess the power quality of the local demand-side grids. The electrical information is analyzed and offered to customers. The customers can control and configure the loads and power supplies through the two-way network. A general measurement procedure in CPES is depicted in Figure 2.

However, as the network scale grows, the complexity of sensing network communication increases. The bandwidth of the network limits the data transition capability. The relay mechanism inhibits the packet loss rate and improves the communication reliability; however, communication delays of data packages are getting larger. This leads to the late catch up of the electrical information of the grid at the data management center. Thus, the data management center has to carry on electrical information analysis with incomplete data. This will bring error to the estimation of the state of the grid. When the delayed data package arrives, these out-of-sequence data should be able to compensate the form estimation and help improve the measurement accuracy. In a large-scale electrical monitoring network, out-of-sequence measurement is a common phenomenon and hinders electrical information analysis, which relies on the correct time series data. Figure 1 indicates that at each data fusion point, the collected data will not follow the time sequence order. This brings temporal error in harmonic analysis for the fusion data not in the same temporal section. Power quality assessment requires high confidence, real-time response and secure data transmission. However, out-of-sequence measurement is a vital challenge to this under the distributed measuring architecture. The harmonics is significant for power quality monitoring on the demand side, since it is the key characteristic in analyzing the application sources, fault localization, smart power management and other applications. Harmonic measurement methods have long been studied. The harmonic measurement deployed at each single metering unit can utilize ESPRIT (Estimation of Signal Parameters via Rotational Invariance Technique) , ADALINE (Adaptive Linear Neuron network) , the Kalman filter, etc. [38,39,40]. At the data management center, harmonic measurement results from numerous end devices needing to update, and the transmission delay affects the fusion and analysis accuracy. Harmonic measurement in a distributed network requires precise temporal section error and phase metering among various harmonics. The 1-μs temporal error between sensing nodes leads to the $1.08^{\prime }$ bias of the baseband phase, which does not fulfill the standard harmonic measuring requirement in IEC 61000-3-2-2002.

Beyond harmonic measurement, the analysis process at the data management system is also affected. Harmonic identification is important in harmonic analysis and widely applied in safety monitoring and assessment of the smart grid. Harmonic identification relies on the analysis of a time sequence of multi-channel electrical data and is affected by out-of-sequence measured data. In this paper, harmonic source identification is settled to evaluate the influence of the OOSM problem. The harmonic sources S and measurement data X follow:

(1) X=AS+N

$X\in R^{M\times T}$, containing *M* channels and *T* time points. $S\in R^{N\times T}$, and *N* is the number of sources. $A\in R^{M\times N}$ is the measuring matrix, and N is the distorted signal. The identification of the harmonic sources from the measurement data is a optimization problem of the following kind:

(2) $$\hat{S}=\mathrm{WX}=A^{-1}X$$

The W is the transfer matrix. $\hat{S}$ is the estimate sources using independent component analysis. A widely-applied method for harmonic identification is independent component analysis (ICA); its principle and realization have been explained in [41]. In this paper, this method is utilized to testify to the effects of disordered sequences on harmonic source identification accuracy.

In addition, for online harmonic analysis and identification, electrical data of the grid are transmitted, which increase the communication load. This leads to a multi-step lag in multi-channels and results in larger temporal errors. In this paper, compressive sensing is brought in to decrease the possible lags. The harmonic analysis schedule is shown in Figure 2. Through the distribute sensing network, the asynchronous measurement data are regulated by an OOSM filter. The measurement data in order are then used for harmonic analysis.

### 2.2. Out-of-Sequence Measurement Problem Formulation

The harmonic measurement is under a distributed multi-channel network fulfilling the following assumptions:

**Assumption** **1.**  *All of the electrical measure nodes in the network are identical and perform asynchronous measurement.*

Without loss of generality, an electrical measuring device is mentioned in the following section as sensing node in a distributed sensor network. This assumption guarantees a homogeneous sensing environment. The hybrid sensor network leads to disorders among heterogeneous sensing data, which is not considered in this paper.

**Assumption** **2.**  *Temporal deviations among the multiple channels of sensing nodes are introduced in the asynchronous measurement of each node and transmission latencies.*

**Assumption** **3.**  *The measurement process is in distributed sensing nodes, and data fusion is deployed in an integrated cluster head.*

Considering multiple channel data measurement and transition under large-scale sensor network. In the *i*-th channel, if electrical data at $t_{\kappa }$ take a longer time to reach the fusion node, these out of sequence data are represented as x(κ). In the measuring sequence, k−l<κ<k−l+1, and x(κ) arrives at the fusion node only for the estimation of x(k); the fusion process is in sequence before x(k−1), and at the x(k), there exists an *l*-step lag OOSM to be corrected. If l=1, x(κ) is only one step behind x(k). $l_{max}$ is the largest disorder of the sequence. In a network of *N* sensor nodes, the nonlinear dynamic function possesses the following form:

(3) $$x\left(k\right)=F\left(x\left(k-1\right)\right)+w\left(k,k-1\right)$$

where $x\left(k\right)=\left[x_{1},x_{2},\ldots ,x_{N}\right]$ is the measured data from *N* sensor nodes at time $t_{k}$ while k=1,2,…; F(·) is the nonlinear state transition matrix. w(k,k−1) is a zero mean Gaussian white noise at the *i*-th channel. The measurement equation of the electrical sensing network is:

(4) $$z\left(k\right)=H\left(x\left(k\right)\right)+v\left(k\right)$$

For electrical harmonics, a linear approximation of the kinematic function of the voltage waveform is proposed in [42]:

(5) $$\begin{array}{ccc} z(k) & = & \sum A_{i}\sin\left(\omega_{i}t_{k}+\phi_{i}\right)+v\\ & = & \sum A_{i}\left(\sin\omega_{i}t_{k}\cos\phi_{i}+\cos\omega_{i}t_{k}\sin\phi_{i}\right)+v \end{array}$$

Define $x_{i}$ as:

(6) $$\begin{array}{cc} x_{2p-1}\left(k\right)= & A_{p}\sin\omega_{p}t_{k} \end{array}$$

(7) $$\begin{array}{cc} x_{2p}\left(k\right)= & A_{p}\cos\omega_{p}t_{k} \end{array}$$

Adopt Equation (6) in Equation (5); the measurement equation can be depicted as:

(8) $$x\left(k+1\right)=\left(\begin{array}{ccc} T_{1} & \cdots & 0\\ \vdots & \ddots & \vdots \\ 0 & \cdots & T_{p} \end{array}\right)x\left(k\right)+w\left(k\right)$$

where $T_{i}$ represents the rotation matrix decided by the *i*-th harmonic phase $\phi_{i}$. The measurement matrix is [43]:

(9) $$\hat{z}\left(k\right)=\left[1,1,\ldots ,1\right]\cdot \hat{x}\left(k\right)+v$$

For electrical parameter measurement, the measuring transition matrix H is usually regarded as an identity matrix, which cannot be guaranteed in the practical environment. The out-of-sequence measurement problem occurs in the multi-channel sensing process as shown in Figure 1. All of the sensing channels send their measurement results to the fusion center with their own disorders (Sensor 1 has a four-lag disorder at $t_{2}$, and Sensor 2 has a one-lag disorder at $t_{6}$). For an *l*-lag disorder at $t_{\kappa }$ for the *i*-th sensor node where k-l≤κ≤k, before the $x_{\kappa }$ arrives at the fusion center, to get the real measurement result $x_{k}$, it is necessary to retrodict the ${\hat{x}}_{\kappa }$. From Equation (3), it can be derived that:

(10) $$\hat{x}\left(\kappa \right)=F\left(\kappa ,k-1\right)x\left(k-1\right)+W\left(\kappa ,k-1\right)$$

Filtering algorithms, such as the Kalman filter, particle filters and artificial intelligent models, have been presented to get the actual measure result x(k) form the past measure result *z*. Covariance of the retrodict result $P\left(k|\kappa \right)=cov[x\left(k\right)|Z^{\kappa }]$ to evaluate the performance. Calculate the filter gain for updating the state x(k) with the earlier measurement z(κ). Update the state estimate x(k|k) to $\hat{x}\left(k|\kappa \right)$, and calculate the corresponding covariance. However, the dynamics of time-varying harmonic does not follow a certain distribution. Changes of the harmonic are driven by customer behaviors. Customers change the states of loads in the grid according to demand. In industry, for instance, the harmonics of numerical control machines change according to the manufacturing schedule. Thus, the harmonics is more like a hidden Markov process. The Monte Carlo process does not describe the process well. Thus, the model in the OOSM algorithm is the key feature.

The basic steps of OOSM are presented in [31]:

Prediction: combine the evolution model $p(x_{k}|x_{k-1})$:
  (11) $$p\left(x_{k}|Z^{k-1}\right)=\int p\left(x_{k}|x_{k-1}\right)p\left(x_{k-1}|Z^{k-1}\right)dx_{k-1}$$
  where
  (12) $$Z^{k}={\left\{z\left(i\right)\right\}}_{i=1}^{k}$$
Filtering: the filtering density $p(x_{K}|Z^{k})$ is obtained by combining the sensor model and the prediction density:
  (13) $$p\left(x_{k}|Z^{k}\right)=\frac{p\left(Z_{k},m_{k}|X_{k}\right)p\left(x_{k}|Z^{k-1}\right)}{\int p\left(Z_{k},m_{k}|X_{k}\right)p\left(x_{k}|Z^{k-1}\right)dx_{k}}$$
Retrodiction: the retrodiction density $p(x_{l}|Z^{k})$ is obtained by combing the object evolution model with the previous prediction and filter densities:
  (14) $$p\left(x_{l}|Z^{k}\right)=\int \frac{p\left(x_{l+1}|x_{l}\right)p\left(x_{l}|Z^{l}\right)}{p(x_{l+1}|Z^{k})}dx_{l+1}$$

Traditional OOSM algorithms are based on the Kalman filter or the particle filter with statistic theory [7]. In the random-lag, multi-channel situation, a priori dynamic function F is necessary, and statistics analysis is comprehensive and difficult to implement. For target tracking, the dynamics can be regulated as certain patterns, like uniform rectilinear motion or constant acceleration. However, in the residential and industrial power utilization network, the customer devices are becoming the main part of the harmonic sources. The harmonics injected into the grid depends on the user behavior, which is difficult to predict. The harmonics does not change in a definite pattern. Online identification of the non-stationary harmonic sources requires a real-time and precise measurement. Thus, the out-of-sequence problem is not ignorable in the problem, and an optimized estimation method is required.

## 3. Data-Aware Retrodiction for Out-of-Sequence Measurement

### 3.1. Harmonic Modeling Based on a Nonlinear Autoregressive Exogenous Model Neural Network

**Figure 3:**
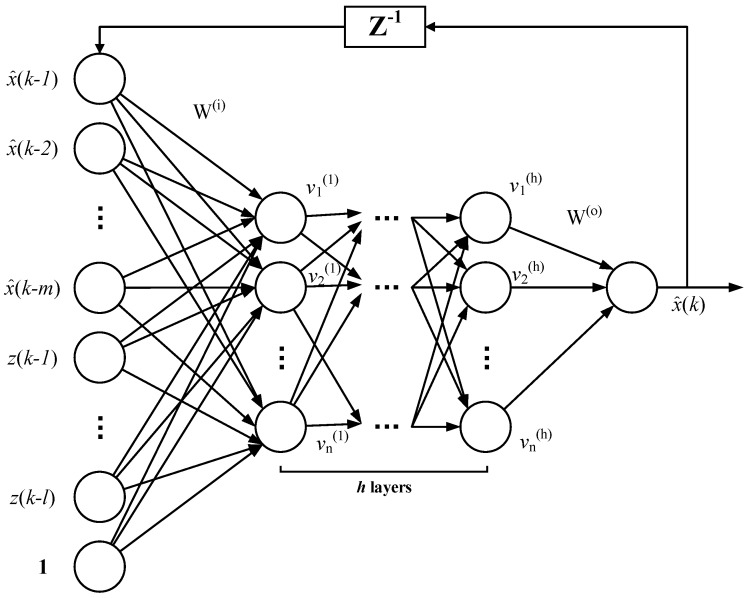
Parallel nonlinear autoregressive model with exogenous inputs (NARX) structure with *h* hidden layers and recursive feedback. The nodes of each hidden layer are *n*. *d* is the output estimated data in the time sequence, and *m* is the output depth. *u* is the measured input data in the time sequence, and *l* is the input depth. The maximum delay of the out-of-sequence $l_{max}$ should be no more than *l*.

Specific nonlinear models for electrical parameters are difficult to describe. The artificial neural network has gained the reputation of approximating nonlinear systems and does not depend on prior models. There has been research on harmonic estimation and prediction based on artificial neural networks [29,30]. The nonlinear autoregressive model with exogenous inputs (NARX) has been exploited for dynamic system prediction in financial, engineering, medical areas, etc. For OOSM in harmonic measurement among sensor networks, the disordered dynamic system can be predicted and rectified through the NARX neural network. In [28], the NARX neural network is utilized to solve the OOSM problem. Since the disorder measurement sequence is the result of the high communication delay, Liu used a compressive sensing method to reduce the communication requirements. Thus, the multi-lag disorder phenomenon will decease to one-lag disorder. Then, the NARX neural network adjusts the one-lag OOSM. However, the multi-lag disorder is non-neglectable as the network scale grows. Thus, an NARX-based method for multi-lag OOSM is proposed in the following. The NARX model is described in Figure 3.

(15) $$\begin{array}{cc} v^{\left(1\right)}\left(k\right)= & f(W^{i}\cdot u) \end{array}$$

(16) $$\begin{array}{cc} v^{\left(j\right)}\left(k\right)= & f[W^{\left(j\right)}v^{\left(j-1\right)}\left(k\right)],2\leq j\leq h \end{array}$$

(17) $$\begin{array}{cc} \hat{x}\left(k\right)= & f[W^{o}v^{\left(h\right)}\left(k\right)] \end{array}$$

where u represents the input vector [$\hat{x}\left(k-1\right),\hat{x}\left(k-2\right),\ldots ,\hat{x}\left(k-m\right),z\left(k-1\right),z\left(k-2\right),\ldots ,z\left(k-l\right)]$. *h* is the number of hidden layers, and $v^{\left(i\right)}$ denotes the parameters of the *i*-th hidden layer. $W^{i}$ is the weight of input layer and $W^{o}$ the output layer. Equation (15) is the relationship between the input layer u and the first hidden layer $v^{\left(1\right)}\left(k\right)$ at $t_{k}$. Equation (16) is the relationship between the hidden layers. Equation (17) describes the relationship between the last hidden layer $v^{\left(h\right)}\left(k\right)$ and the output layer $\hat{x}\left(k\right)$ at $t_{k}$. As for the whole network, the relationship between the input and output can be described as:

(18) $$\begin{array}{cc} \hat{x}\left(k\right)= & G[\hat{x}\left(k-1\right),\hat{x}\left(k-2\right),\ldots ,\hat{x}\left(k-m\right),\\ & z(k-1),z(k-2),\ldots ,z(k-l)] \end{array}$$

where z is the original measurement harmonic data and $\hat{x}$ is the prediction of the electrical parameters at the next time point, to occupy the vacancy of datasets to be analyzed. l>0 and m>0 are the input and output scales, respectively. Function G(·) is the retrodiction transfer function, which is to be approximated by the *h* layer feedforward network of function f(x).

The NARX network has two model types: the serial and parallel mode [44]. The serial mode calculates the $\hat{x}\left(k\right)$ according to the definite measured data, namely as the Equation (18) shows. The output can be written in the following form:

(19) $$\begin{array}{cc} \hat{z}\left(k\right) & =G[\hat{x}\left(k-1\right),\hat{x}\left(k-2\right),\ldots ,\hat{x}\left(k-m\right)\\ & d(k-1),d(k-2),\ldots ,d(k-l)] \end{array}$$

where d(k) represents the actual output data, while $\hat{x}\left(k\right)$ is the estimated result at the *k*-th time point. In the neural network, the outputs of the hidden layers are:

(20) $$\upsilon_{p}\left(k\right)=G\left[\sum_{q=1}^{l+m+1}W_{pq}^{\left(1\right)}x_{q}\left(k\right)\right]$$

where $\upsilon_{p}\left(k\right)$ is the neuron in the *p*-th hidden layer. $W_{pq}^{\left(i\right)}$ is the weight connecting the *q*-th input and *p*-th neuron in the *i*-th hidden layer. Thus, the network output is:

(21) $$\hat{z}\left(k\right)=\sum_{p=1}^{n}W_{p}^{\left(0\right)}\upsilon_{p}\left(k\right)$$

The parallel model utilizes recent estimation results for estimation at the *k*-th time point:

(22) $$\begin{array}{cc} \hat{z}\left(k\right) & =G[\hat{x}\left(k-1\right),\hat{x}\left(k-2\right),\ldots ,\hat{x}\left(k-m\right)\\ & z(k-1),z(k-2),\ldots ,z(k-l)] \end{array}$$

In this paper, the parallel NARX structure is adopted for prompt feedback, as shown in Figure 3. Each layer uses the sigmoid function as f(x), for the sake of its low computational complexity on derivation. $f^{\prime }\left(x\right)=f\left(x\right)f\left(1-f\left(x\right)\right)$, which is useful in the following analysis.

The training method uses the Levenberg–Marquardt back propagation algorithm, which is a modification of the Gauss–Newton method; the updating equation has the following form:

(23) $$w_{n+1}=w_{n}-{\left(J_{w}{\left(n\right)}^{T}J_{w}\left(n\right)+\mu I\right)}^{-1}J_{w}{\left(n\right)}^{T}e\left(n\right)$$

where $w_{n}$ denotes the weights of each layer in the *n*-th iteration. $J_{w}\left(n\right)$ is the Jacobi matrix of this layer, which can be defined as $J_{w}\left(n\right)={\left\{\partial e/\partial w\right\}}_{ij}$. e(n) is the error in each layer. At the output layer, $e^{o}\left(n\right)={\left(\hat{x}\left(n\right)-z\left(n\right)\right)}^{2}/2$. At the hidden layer, the error is defined as the difference of each layer output.

The NARX neural network is an instance of a recurrent neural network. The multi-hidden layers can describe the dynamic feature of signals in various scales and the hidden Markov process of the customer event. Although the NARX neural network is dynamic over time, by introducing the time point as input, the temporal feature can be detected in the NARX model.

### 3.2. Data-Aware Retrodiction Based on the NARX Neural Network

From the description of the OOSM method in Section 2, when the out-of-sequence data arrive, the prediction and filtering process can rely on the model in the neural network. The retrodiction process is the main task in updating the latest harmonic estimation. When the measured data at $t_{\kappa }$ are missing, the input data lack the $z_{\kappa }$. This can be replaced with the estimation $\hat{x_{\kappa }}$ in the last iteration. According to the back propagation method in Equation (23), the updating of the matrix has the following form. Define $S\left(n\right)={\left(J_{w}{\left(n\right)}^{T}J_{w}\left(n\right)+\mu I\right)}^{-1}$; thus:

(24) $$\begin{array}{ccc} \Delta w^{o} & = & S^{o}\left(\kappa \right)J_{w}^{o}{\left(n\right)}^{T}e\left(\kappa \right) \end{array}$$

(25) $$\begin{array}{ccc} \Delta w^{h} & = & S^{h}\left(\kappa \right)J_{w}^{h}{\left(n\right)}^{T}e\left(\kappa \right) \end{array}$$

(26) $$\begin{array}{ccc} \Delta w^{i} & = & S^{i}\left(\kappa \right)J_{w}^{i}{\left(n\right)}^{T}e\left(\kappa \right) \end{array}$$

$w^{o},w^{h},w^{i}$ are the weights in the output layer, hidden layer and input layer, respectively. Δw is the weight updating. When the out-of-sequence data $z_{\kappa }$ arrive at $t_{k}$, it shall update the input layer $u_{k}$. Thus, the out-of-sequence lag l=k−κ. Define $\Delta x=z_{\kappa }-{\hat{x}}_{k}$, and the change is trivial compared to $z_{\kappa }$; thus, if operating an identical neural network offline with the in-sequence data, the updating weights should change in Equation (26), updating the input elements *u* with $z_{\kappa }$. Thus, at $t_{k}$, the distortion in weights by Δx should be verified. Considering that it is clear that the estimation $\hat{x_{\kappa }}$ will bring in errors, the training algorithm can change from the Levenberg-Marquardt method to the gradient method, which has a lower convergence rate. This reduces the impact of the estimation error to the network. The gradient algorithm has the following form:

(27) $$\Delta w=\eta \frac{\partial e}{\partial w}$$

The input error Δx expands to the feedforward network. For the neural network described in Figure 3, the first hidden layer output has the following form:

(28) $$v=f(W^{\left(i\right)}u)$$

The error introduced by Δx can be derived as:

(29) $$\Delta v^{\left(1\right)}=f\left(W^{\left(i\right)}\left(u+\Delta u\right)\right)-f\left(W^{\left(i\right)}u\right)=J_{x}^{\left(1\right)}\Delta u$$

where the Jacobi matrix is:

(30) $$J_{x}^{\left(1\right)}=\left\{\partial f\left(W^{\left(i\right)}x\right)/\partial x_{i}\right\}=diag\left(f^{\prime }\left(W^{\left(i\right)}x\right)\right)W^{\left(i\right)}$$

In other layers, the input errors have a recursive form:

(31) $$\begin{array}{ccc} \Delta v^{\left(i\right)} & = & (\prod_{j=1}^{i}J_{x}^{\left(j\right)})\Delta u \end{array}$$

(32) $$\begin{array}{ccc} \Delta o & = & J_{x}^{\left(o\right)}\left(\prod_{j=1}^{h}J_{x}^{\left(j\right)}\right)\Delta u \end{array}$$

Thus, the output error magnifies the input error through each layer. The backward feedback on the weights modification depends on the output error and goes back though the network. For gradient backpropagation algorithm, the updating functions of weights in each layer are:

(33) $$\begin{array}{ccc} \Delta w_{m,n}^{o} & = & \eta \frac{\partial e(\kappa )}{\partial w_{m,n}^{o}}=\eta \left(z-\hat{x}\right)f^{\prime }\left(w^{o}v^{\left(h\right)}\right)v_{n}^{\left(h\right)}=\eta \delta_{m}^{o}v_{n}^{\left(h\right)} \end{array}$$

(34) $$\begin{array}{ccc} \Delta w_{m,n}^{\left(i\right)} & = & \eta \frac{\partial e(\kappa )}{\partial w_{m,n}^{\left(i\right)}}=\eta \left[\sum_{k=1}w_{km}\delta_{m}\right]f^{\prime }\left(w^{\left(i\right)}v^{\left(i\right)}\right)v_{m,n}^{\left(i\right)}=\eta \delta_{m}^{\left(i\right)}v_{n}^{\left(i-1\right)} \end{array}$$

(35) $$\begin{array}{ccc} \Delta w_{m,n}^{i} & = & \eta \frac{\partial e(\kappa )}{\partial w_{m,n}^{i}}=\eta \left[\sum_{k=1}w_{km}\delta_{m}\right]f^{\prime }\left(w^{i}u\right)u_{n}=\eta \delta_{m}^{i}u_{n} \end{array}$$

where *δ* represents the error in each layer. For the output layer, the change of the input brings the weight updating error:

(36) $$\delta w_{m,n}^{o}=\eta \left(z-\hat{x}-\Delta o_{m}\right)f^{\prime }\left(w^{o}\left(v^{\left(h\right)}+\Delta v^{\left(h\right)}\right)\right)\left(v_{n}^{\left(h\right)}+\Delta v_{n}^{\left(h\right)}\right)-\Delta w_{m,n}^{o}$$

Neglecting the higher order of Δ, the formulation can be derived as:

(37) $$\delta w_{m,n}^{o}=\eta \left(z-\hat{x}\right)f^{\prime }\left(w^{o}v^{\left(h\right)}\right)\Delta v_{n}^{\left(h\right)}+\eta f^{\prime }\left(w^{o}\left(v^{\left(h\right)}\right)\right)v_{n}^{\left(h\right)}\Delta o_{m}$$

It can be figured out that the change of the weights is composed of the errors of both the output layer and the input layer. In the back propagation method, the weights update in a recursive manner; thus, the estimation error can be traced down by updating the updating character *δ* in each layer. In Equation (33), the changes of elements in each layer are verified in Equations (29) and (31). For *δ*, the changes are:

(38) $$\begin{array}{ccc} \Delta \delta_{m}^{\left(i\right)} & = & \left[\sum_{k=1}w_{km}\Delta \delta_{m}^{\left(i+1\right)}\right]f^{\prime }\left(w^{\left(i\right)}v^{\left(i\right)}\right) \end{array}$$

(39) $$\begin{array}{ccc} \Delta \delta_{m}^{i} & = & \left[\sum_{k=1}w_{km}\Delta \delta_{m}^{\left(1\right)}\right]f^{\prime }\left(w^{i}u\right) \end{array}$$

Thus, the weight updating errors for hidden layers and the input layer are:

(40) $$\begin{array}{ccc} \delta w_{m,n}^{\left(i\right)} & = & \eta \delta_{m}^{\left(i\right)}\Delta v_{n}^{\left(h\right)}+\eta \Delta \delta_{m}^{\left(i\right)}v_{n}^{\left(h\right)} \end{array}$$

(41) $$\begin{array}{ccc} \delta w_{m,n}^{i} & = & \eta \delta_{m}^{\left(1\right)}\Delta u_{k}+\eta \Delta \delta_{m}^{\left(1\right)}u_{k} \end{array}$$

From the above, the estimation error Δx propagates forward from the input layer to the output layer, changing the elements in each layer. Then, the back propagation method propagates the error backward to the layer weights. When the measured data z(κ) arrive, the estimation error Δx can be calculated. The bias of weights introduced by the estimation error can be rectified by retrodiction. The retrodiction method is described below.

#### 3.2.1. Single-Lag Out-of-Sequence Measurement Solution

In the single-lag situation, the estimation error $\Delta x=z\left(\kappa \right)-\hat{x}\left(\kappa \right)$ at the input layer has propagation only once in the network. $\Delta u\left(k-l\right)={\left[\Delta x,0,0,\ldots \right]}^{T}$. The estimation error on the weights can be rectified backward. The single-lag OOSM algorithm is listed as follows.

Step 1: With Δu, calculate the errors of the elements in each layer through Equations (29)–(32).
Step 2: Calculate *δ* and Δδ in each layer by Equations (33)–(39).
Step 3: Calculate the error of weights with Equations (40) and (41).
Step 4: Update the weights with:
  (42) $$\begin{array}{ccc} {\bar{w}}_{m,n}^{o} & = & w_{m,n}^{o}-\delta w_{m,n}^{o} \end{array}$$
  (43) $$\begin{array}{ccc} {\bar{w}}_{m,n}^{\left(i\right)} & = & w_{m,n}^{\left(i\right)}-\delta w_{m,n}^{\left(i\right)} \end{array}$$
  (44) $$\begin{array}{ccc} {\bar{w}}_{m,n}^{i} & = & w_{m,n}^{i}-\delta w_{m,n}^{i} \end{array}$$
Step 5: Update the estimation of $\hat{x}\left(k\right)$.

#### 3.2.2. Multi-Lag Out-of-Sequence Measurement Solution

In an *l*-lag out-of-sequence measurement circumstance (l>1), the estimation ${\hat{x}}_{\kappa }$ stays in the input layer until $l>l_{max}$. As long as ${\hat{x}}_{\kappa }$ is in the input layer, it recursively introduces the deviations in each iteration. In the first iteration, the input deviation is $\Delta u\left(k-l\right)={\left[\Delta x,0,0,\ldots \right]}^{T}$. In the second iteration, when the input deviation has already propagated to the output layer, the deviation of the new estimation of $\hat{x}\left(k-l+1\right)$ is Δo(k−l) as in Equation (32). Thus, the input deviation is $\Delta u\left(k-l\right)={\left[o(k-l),\Delta x,0,\ldots \right]}^{T}$. Since this NARX neural network has only one output element, Δo(k−l) can be expressed more concisely. Define $S_{x}\left(k\right)=J_{x}^{\left(o\right)}\left(k\right)\prod_{j=1}^{h}J^{\left(j\right)}{\left(k\right)}_{x}$, and construct the matrix:

(45) $$S_{x}\left(k\right)=\left(\begin{array}{cccc} S_{x}\left(k\right) & 1 & & \\ & \ddots & \ddots & \\ & & \ddots & 1\\ & & & S_{x}\left(k\right) \end{array}\right)$$

Thus, in the *p*-th iteration, when $z_{\kappa }$ arrives, the input layer deviation is:

(46) $$\Delta u\left(k-l+p\right)=\left(\prod_{i=1}^{p}S_{x}\left(k-l+i\right)\right)\Delta u\left(k-l\right)$$

If the NARX neural network already convergences to a certain model, the deviation is trivial compared to the input data. For a suboptimal approximation, the retrodiction will not change the latest weights and only adjust the output. The weights will be updated in the following measurement process when new data enter. To reduce the error, the rectify process for single-lag out-of-sequence measurement is repeated *l* times recursively. The multi-lag out-of-sequence measurement method is listed as follows:

Step 1: Calculate $\Delta x=z\left(\kappa \right)-\hat{x}\left(\kappa \right)$, and initialize $\Delta u\left(k-l\right)={\left[\Delta x,0,0,\ldots \right]}^{T}$, p=0;
Step 2: Perform the single-lag OOSM algorithm with Δu(k−l+p), and set p=p+1;
Step 3: Update $\Delta u\left(k-l+p\right)=S_{x}\Delta u\left(k-l+p-1\right)$;
Step 4: Go back to Step 2 until p=l−1.
Step 5: Update the latest estimation $\hat{x}\left(k\right)$.

Thus, the retrodiction of the time sequence of electrical harmonics is reached following the recursive process of the feedback neural networks. The weights are self-regulated as the temporal data sequence going through the network. The OOSM process is data-aware and should uniformly converge to the practical model of electrical harmonics. Deploying the retrodiction method in a cyber-physical energy system described in Section 2, the distributed harmonic measurement is scheduled as follows:

Step 1: Sensor nodes of the electric monitoring network measure the harmonics in each branch. The harmonic measurement algorithm is ADALINE [45]. The distributed nodes calculate the amplitudes and phases of each order of harmonics in their branches and send the harmonic data up to the data management system through the two-way sensing network.
Step 2: Upon the arrival of the harmonic information of the network, the data management system updates the cached electrical state. If harmonic data of certain branches are late, update the harmonic parameter with the former estimate value and go on measuring.
Step 3: When the out-of-sequence measurement arrives, the OOSM algorithm retrodicts the transmission error of the end notes using the multi-lag out-of-sequence measurement method and updates the latest estimation of the harmonic information of the grid.
Step 4: The harmonic analysis applications, such as harmonic sources identification, are processed with the updated harmonic estimation result.

In this case, the retrodiction algorithm is embedded in the harmonic measurement process without interfering with the normal detection of the monitoring process. Different from [28], the compressive sensing process is abandoned, since the reconstruction algorithm is of high computational complexity. The multi-lag OOSM method should be able to offer sufficient precision. The influence of the algorithm on the monitoring network is evaluated in the following.

### 3.3. Evaluation of Data-Aware Retrodiction for Harmonics Measurement

The retrodiction algorithm can be implemented in the centralized computing unit or distributed data fusion units as long as the out-of-sequence data occur. Other than the measurement accuracy, computational complexity and memory consumption are important indexes indicating the algorithm performance. This is important for online harmonic analysis applications. This section compares to the computational complexity and memory consumption of the NARX-based method with the Kalman filter- and the particle filter-based methods.

#### 3.3.1. Computational Complexity Evaluation

Computational complexity guarantees the implementation of real-time data analysis over the network. The computational complexity of the Kalman filter-based OOSM is presented in [46]. In general, the computation complexity of the neural networks is exponential and is decided by the layers, input numbers and neural network type. Define $n_{x}$ as the number of inputs, *h* as the numbers of hidden layers, $p_{i}$ as the number of elements in the *i*-th hidden layer, *m* as the number of measurements and $N_{w}$ as the number of total weights. As long as the number of elements in the hidden layers is no less than the input layer, which is normal, then $N_{w}=n_{x}p_{1}+p_{1}p_{2}+p_{2}\approx hn_{x}^{2}$. When an *l*-lag out-of-sequence measurement occurs, the retrodiction algorithm contains *l* cycles of forward and backward propagation. According to [47], the gradient backpropagation algorithm’s computational complexity is $O(N_{w})$. Forward propagation is also a $O(N_{w})$ computation. For the NARX neural network-based retrodiction method, the computational complexity is $O\left(lN_{w}\right)\approx O\left(lhn_{x}^{2}\right)$.

The computational complexity of the Kalman filter- and the particle filter-based methods are investigated, as well. Kalman filter-based methods include Bl [9], Lanzkron [12], ALG-S [13] and ALG-I [14]. Particle filter-based methods include A-PF [17] and SERBPF [21]. The computational complexity information of the Kalman filter-based methods is from [48], and that of particle filter-based methods is from [17,21]. The results are simplified to express the level of the computational complexity, rather than the exact computational consumption. The comparison of the computational complexity of various OOSM methods is displayed in Table 1.

Apparently, delay order, input element number and maximum permitted delay are the main factors for the computational complexity of the results. Kalman filter-based methods (Bl, Lanzkron, ALG-S and ALG-I) offer a calculation consumption of $O(ln_{x}^{3})$, while particle filter-based methods (A-PF and SERBPF) provide a complexity of $O(n_{x}^{2})$ or less. SERBPF optimizes the calculation and deceases the calculation consumption. Computational complexity of the NARX-based method relies more on the neural network structure. If the number of hidden layers $p_{i}\geq n_{x}$, thus the NARX-based method offers $O(n_{x}^{2})$ at best, which is at the same level as A-PF. To reduce the computational complexity, the network should be a tradeoff between the precision and computing requirements.

#### 3.3.2. Memory Consumption

The storage and computational load of the OOSM methods are compared under the same maximum delay *l*. Analyses of the memory consumption of Kalman filter- and particle filter-based methods are in [48]. Define the neural network hidden layer number *h* and number of nodes *n* in each hidden layer. Each prediction assimilates *m* measurements and the input number m+k. Discarding the training consumption, the retrodiction process relies on the network. The memory consumption of NARX is the weights and nodes of the neural networks. Input weight W(i) is (m+l)n, and in each layer, the hidden layer represents $n^{2}$ weight variables. With the hn+(m+l) nodes, the memory consumption is $\left(m+l\right)\left(n+1\right)+hn^{2}+hn$. Assuming that *n* and $n_{x}$ are in the same order of magnitude, the memory consumption can be estimated with $n_{x}$. The memory consumptions of various algorithms are shown in Table 2. OOSM algorithms based on the Kalman filter (Bl, Lanzkron, ALG-S, ALG-I) and the particle filter (A-PF, SERBPF) are compared. The data source is the same as the articles mentioned above. It can be seen that the memory consumption of the NARX-based method is remarkable among these OOSM methods. This is because the proposed method builds the harmonic model through an artificial neural network, which inherently occupies a large amount memory. Furthermore, the NARX network aims at approximating the harmonic dynamic model more precisely than the kinematic models in the Kalman filter. Other than the neural network, the OOSM process hardly adds further memory requests.

With the analysis and comparison above, it can be concluded that the NARX-based OOSM method costs $O(n_{x}^{2})$ in memory consumption and $O(n_{x}^{2})$ in the computation cost, which is not outstanding among the algorithm, but provides a reasonable hardware consumption. The NARX algorithm does not need to store the measured history data, and instead, keeps the past information in the network. This provides a reduction of the computational complexity.

Above all, the proposed algorithm offers a reasonable memory and temporal consumption. This is favorable in a distributed sensing network, sparing more information for other applications in cyber-physical systems.

## 4. Experiments and Analysis

### 4.1. Experiment Setup

**Figure 4:**
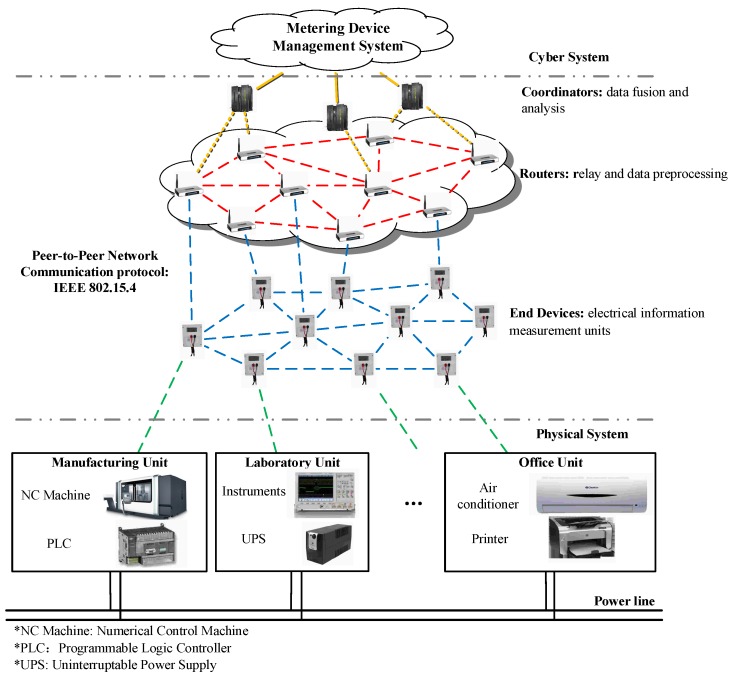
The distributed electrical information monitoring network structure of the cyber-physical energy system testbed in the residential power consumption network.

To evaluate the performance of the OOSM method on the harmonic analysis, a testbed to approximate the cyber-physical energy system in the residential power consumption network is built in this section. A two-way distributed metering framework for electrical information monitoring is deployed. The distributed network sensing architecture is depicted in Figure 2. This network measures and transmits the electrical parameters by multichannels asynchronously. The reconstruction of the signal and harmonic analysis algorithm is realized on the data management center. The power supply is 220 V/50 Hz. The appliances contain linear loads, such as lamp humidities that do not produce harmonics, and nonlinear loads, like rotor rigs, microwaves, air-conditioners, etc., which are demand-side harmonic sources. Nonlinear loads are controlled during the experiment. Switch actions will be detected in harmonic sensing. The Intelligent sensing nodes use ADE7754 to measure electrical parameters with a sampling frequency up to 14 kHz. Signal processing methods are realized on ARM. The electrical characteristics, including voltages, currents, frequency, power, harmonic amplitudes, etc., are reported to the data center. The chip CC2430 realizes data transmission through IEEE 802.15.4 protocols. The data packages follow the protocol standard DL/T 645-2007. The sensing network contains 100 sensing nodes, including 84 end devices, 13 routers and 3 coordinators. An end device calculates and uploads the electrical parameters every second. Then, the router fuses and relays the electrical data measured by end devices to the coordinators. All end devices and routers have the same hardware structure. The coordinators receive the data from the router and send them to the meter data management system. The data management center executes the retrodiction algorithm to correct the electrical data and analyzes the electric information with Intel i5 CPU and 4 G memory. The main structure of the testbed is shown in Figure 4. The harmonic measurement results are compared to the standard Fluke 435. Within the distributed sensing network, the out-of-sequence problem can be observed.

Considering the measurement requirement of different electrical characteristics, harmonic measurement is selected to verify the proposed retrodiction method. In the following, the computation complexity, memory consumption and influence on harmonic measurement applications of the NARX-based OOSM algorithm is compared to other OOSM methods. Time-varying harmonic and transient harmonic measurements are tested. During the measurement process, the Kalman filter and the particle filter need kinematic equations to calculate the estimation of the harmonic. The training method of the NARX-based OOSM method is the same as in Section 3.2, and the training dataset is explained in each case. In these experiments, the Kalman filter and the particle filter use the dynamics described in Equations (8) and (9) [49,50]. Apparently, the kinematic model is linear; the Monte Carlo method in the particle filter can approximate the nonlinear feature of the harmonics.

### 4.2. Measurement Precision Comparison

**Figure 5:**
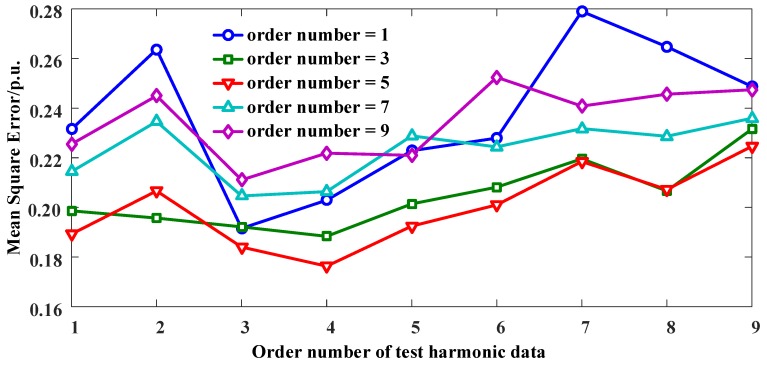
MSE of the out-of-sequence measurement (OOSM) algorithm under various training and test sample sets. Each line represents the results under specific disorder level training data.

The out-of-sequence measurement is implemented on the centralized data management center. To examine the OOSM algorithm’s effectiveness, a simulation experiment is designed with data under various lag levels. Training datasets for the NARX neural network contain 70% of 4-h electrical data of 100 channels, and testing datasets contain another 30% of the electrical data. Based on electrical parameters measured through CPES, multi-lag data sequences can be inserted into the time sequence under a certain frequency through sorting data time sequences. The inserted lags follow a Poisson distribution $\frac{e^{-\lambda }}{k!}\lambda^{k}$. Inserted lags grow from one lag to a maximum of nine lags. The NARX network is trained and tested under various multi-lag levels, which are supposed to turn the disordered data into correct time series. Training and testing datasets from different channels and lag-levels are coupled to examine if the NARX filter trained in a specific dataset is suitable for other lag level data. Average mean square errors (MSE) of the modified data from the original data are recorded to depict the performance of the NARX filter. The results are depicted in Figure 5.

MSEs for the disordered data in the training datasets are between 0.49 and 2.31 under various lag levels. From Figure 5, it can be seen that the MSE reduces between 0.184 and 0.345 after the NARX filter, and the least MSE is reached with 1-lag train datasets and 1-lag disordered data. With the disorder growing, disorder errors display an ascending trend. When the network is trained with multi-lag data, MSE rises, indicating that multi-lag disordered data vary from lag numbers, which is not compatible with the lower lag number situation. The optimal circumstance is the low lag number electrical data series, and the NARX neural network is also trained with the same lag data type. However, in the practical situation, the distortion occurs following an exponential behavior as the scale of the network grows [27]. The main lag number remains 2–3 lags. Thus, the training dataset for the NARX-based OOSM method should be the same practical electrical environment. The flexibility of this method among various electrical situations should be examined in future work.

**Figure 6:**
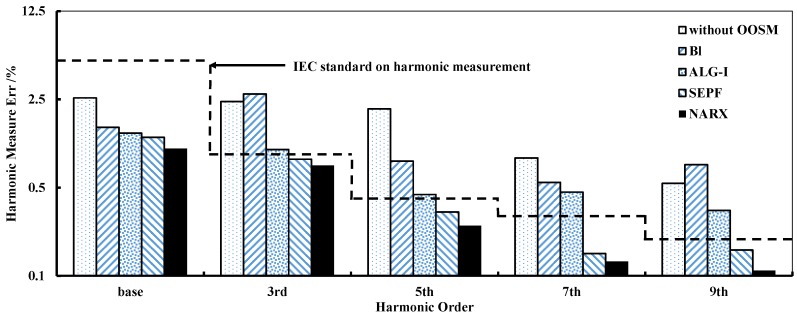
MSE of various harmonic measurements with 4 OOSM algorithms. The dotted line is the upper bound of the harmonic measurement error in the IEC standard 61000-4-7.

Following the harmonic measurement requirements in IEC 61000-4-7, the precision of the base voltage and current is ≤5% of the true values, and the harmonic measurement error limits are: 0.9% for the 3rd harmonic, 0.4% for the 5th harmonic, 0.3% for the 7th harmonic and 0.2% for the 9th harmonic and even harmonics. The NARX-based OOSM method is implemented for harmonic measurements and comparing the MSE with other OOSM methods, including Bl, ALG-I and SEPF. The measurement network scale is 100 nodes, and the average MSE of the harmonic measurement for each OOSM algorithm is shown in Figure 6. The errors of the base current and odd harmonics from the 3rd–9th are tested. The dotted line indicates the requirements for each harmonic order in IEC 61000-4-7-2002. Apparently, without retrodiction, harmonic measurement does not fulfill the standard in a disorder manner. Bl and ALG-I, as suboptimal methods, do not offer a sufficient improvement in precision. SEPF and NARX-based OOSM reach the standard, and the data-aware method maintains a better performance in all orders. The computational complexity of these algorithms is tested in the following.

### 4.3. Computational Complexity Comparison

**Figure 7:**
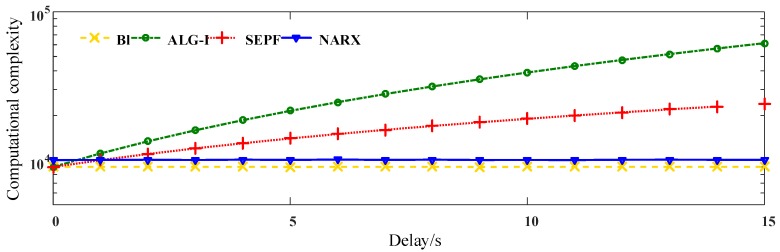
The computational complexity of OOSM methods: Bl, ALG-I, SEPF and NARX based algorithms. The delay of inputs is 5, the computational complexity is represented by the float calculations.

**Figure 8:**
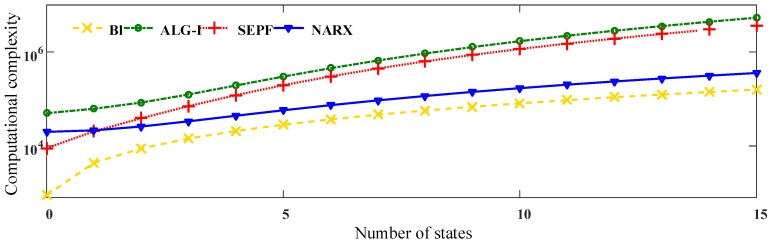
The computational complexity of OOSM methods: Bl, ALG-I, SEPF and NARX-based algorithms. The number of disorders is 9; the computational complexity is represented by the float calculations.

The computational complexity and memory cost of NARX-based OOSM methods are compared to other algorithms. To evaluate the memory and computational performance of the NARX-based method, Kalman filter-based methods, Bl and ALG-I, and the particle filter-based method, SEPF, are selected to compare. The 4 algorithms are tested under the datasets of electrical harmonics. The out-of-sequence harmonic data are measured on the electrical network. The measuring length *N* is 256; the frequency resolution $\Delta_{f}=f_{s}/NP=4.88$ Hz <5 Hz, which meets the requirements in IEC 61000-4-7-2002. The largest disorder of the harmonic sequence is 7. The calculation is implemented on MATLAB 2013b. The harmonic is sorted with the time stamps to be the correct target. The OOSM method is regarded as valid with respect to the MSE of the reordered data. The results of the computational complexity according to the delay length are shown in Figure 7, and those according to the number of states are shown in Figure 8.

From the results, it can be figured out that ALG-I performs the largest number of float calculations. As a suboptimal method, although Bl fails to offer a sufficient harmonic measuring precision, it provides the least computational consumption among the algorithms. NARX-based OOSM behaves more stably than the particle filter-based method SEPF. The NARX-OOSM does not increase the computational capacity on different delays, but increases as the input data grow. However, among the 4 algorithms, NARX managed to maintain a relatively low computation consumption, and the practical results comply with the theoretical analysis from above. Considering the harmonic measuring precision, the proposed data-aware methods are promising among the 4 algorithms in the multichannel harmonic measurement. Harmonic identification analysis depending on time series will be tested in the following section.

### 4.4. Case I: Data-Aware Retrodiction for Non-Stationary Harmonic Measurement

Harmonic measurement is examined to test the effects of various OOSM algorithms on harmonic analysis. Harmonic measurement is important in harmonic analysis and widely applied in safety monitoring and assessment of the smart grid. Harmonic identification relies on the analysis of a time sequence of multi-channel electrical data and is affected by out-of-sequence measured data. In this section, the non-stationary harmonics are injected in the grid to test the OOSM algorithms. The harmonic sources are inverters in speed-varying rotor machines. The rotation speed ranges of these rotor machines are 0–3000 rpm. The rotor standard power is 2.8 kW at 3000 rpm, and the output power changes over time as the speed changes. These machines are controlled independently and inject unstable harmonics into the grid.

**Figure 9:**
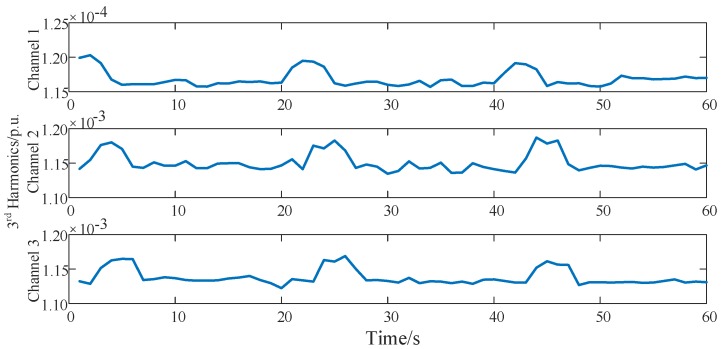
Third current harmonics of 3 typical channels at the same point of common coupling (PCC). The harmonics are independent and non-stationary in all 3 channels.

**Figure 10:**
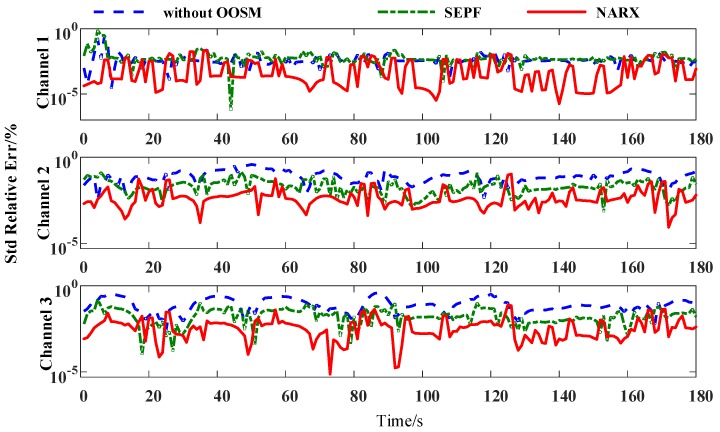
Relative errors of the 3rd harmonic identification with different OOSM methods of different channels.

The harmonic series are measured under the distributed power utilization sensing network. The harmonic measurement algorithm deployed at the end nodes is adaptive linear neuron or, later, adaptive linear element (ADALINE). Data disorders do not exceed 4 lags. Electrical parameters in 100 channels are collected, and 3 channels of the original electrical harmonic current measurement and the harmonic sources are shown in Figure 9. The 3 channels link to the same point of common coupling (PCC), and the harmonic currents of each nonlinear load are tangled with each other in the measurement data. Harmonic measurement results are compared to the sensing results of Fluke 435. Different orders of harmonic currents examine the effects of the OOSM algorithm on harmonic identification. The OOSM improvement in decreasing measurement error is shown in Figure 10, and the MSEs of 100 channels are listed in Table 3.

The 3rd–9th odd harmonics are used for harmonic measurement verification, which are capable of representing the harmonic measurement precision of the OOSM process. The results indicate that the NARX neural network can reduce the error introduced by the out-of-sequence measurement. It can also be figured that the OOSM algorithm alone does not convey a productive decrease on the out-of-sequence measurement error. The MSE of the measurements are reduced by more than 33%.

The retrodiction accuracy of 4 algorithms shown in Table 3 indicates that the NARX-based OOSM method gains a better harmonic measurement result than the other 3 methods, although the deviations are minor. For the 7th and 9th harmonic, the OOSM methods do not make a great difference in accuracy among each other. This indicates that as the magnitudes of the harmonics become small, the effects of the modification are not manifest. The data-aware method’s improvement on non-stationary harmonic measurement is verified.

### 4.5. Case II: Data-Aware Retrodiction for Transient Harmonic Measurement

Transient harmonic measurement is examined in this section to test the effects of various OOSM algorithms on harmonic analysis. The transient harmonics are mainly introduced by switching on or off large nonlinear loads, injecting a large current shock. The transient harmonic source is realized by switching on and off a light wave oven, which injects a 4 A current pulse and lasts for 136 ms. The current injection can be detected from the 3rd harmonic to the 9th harmonic in the channel. Out-of-sequence performance occurs at the time point of the current pulse, and the retrodiction performance is examined by the measurement accuracy of the transient harmonic.

**Figure 11:**
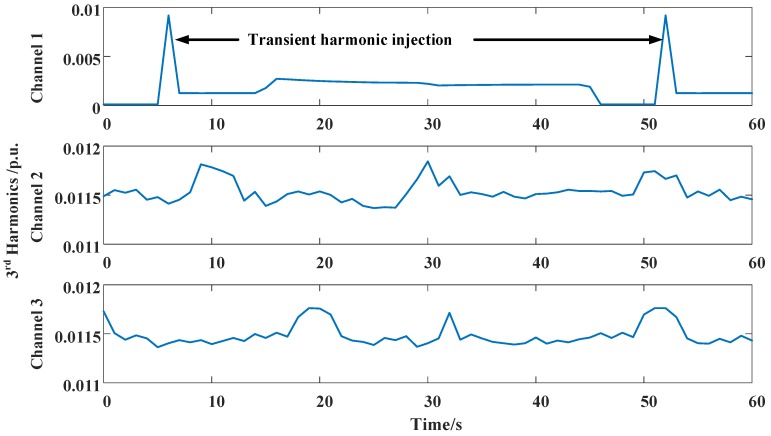
Third current harmonics of 3 typical channels at the same PCC with transient harmonic injection in Channel 1.

**Figure 12:**
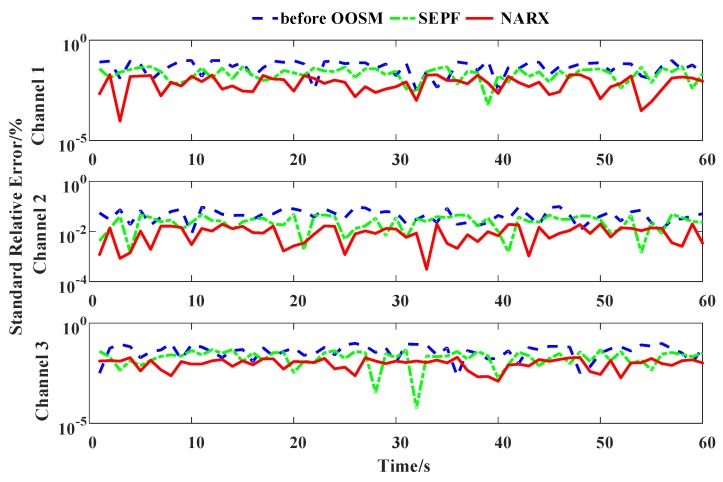
Relative errors of the 3rd harmonic identification with different OOSM methods of different channels.

The harmonics are measured under the same residential sensing network as in Case I, while the transient harmonic source is deployed. There are 100 sampling channels and 3 channels of the original electrical harmonic current measurement, and the harmonic sources are shown in Figure 11. The lines of the 3 channels are linked to the same point of common coupling (PCC). The out-of-sequence phenomena occur during the measurement process, and at the transient harmonic injection, the measurement data are late for 2 periods, namely a 2-lag disorder. Figure 12 shows the harmonic measurement error with and without OOSM methods. SEPF and NARX-based OOSM are compared. The 3rd–9th odd harmonics are used to compare the measurement precision.

When the transient harmonic pulse does not occur in Channel 1, Figure 11 shows that the NARX-based OOSM method leads to a less standard relative error of harmonic measurement than SEPF, as has been discussed in Case I. When the transient harmonic pulse occurs with the 2-lag disorder, the estimation error is remarkable without retrodiction. SEPF and NARX-based OOSM methods both reduce the estimation error, and the NARX-based method offers a better performance. Yet, the estimation error at the transient harmonic event is still larger than other time points. It also shows that the OOSM method performs a larger reduction with frequently changing harmonic waves in Channels 2 and 3. The harmonics in Channel 1 change thoroughly, but smoothly; the OOSM makes a minor fluctuation. This indicates that OOSM modifies greatly instantaneous and fierce harmonic fluctuations, such as voltage sags and instant impacts.

Table 4 lists the MSEs of 100 channels. The results show that among the 4 algorithms, the NARX-based OOSM method gains a better harmonic measurement result than the other 3 methods with frequent transient harmonic injections. The MSEs of the measurements are reduced by more than 24% with frequent transient harmonic events. Yet, OOSM methods do not make a great difference in measurement accuracy for high order harmonics, since their magnitudes are small. Thus, the data-aware method’s improvement on transient harmonic measurement is verified.

### 4.6. Case III: Data-Aware Retrodiction-Based Harmonic Identification

**Figure 13:**
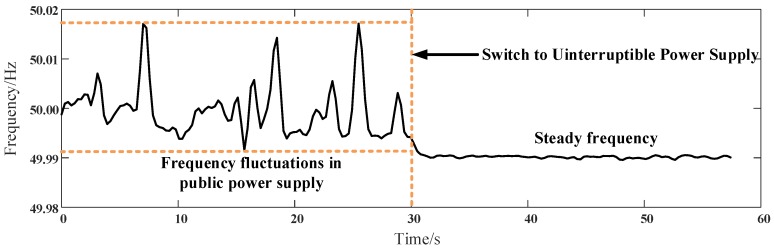
Base frequency changes during the measuring process. During 0–30 s, the network is connected to the public power; during 30–60 s, it is connected to the uninterruptible power supply.

To examine the harmonic measurement in a practical environment, the situation of a power outage is simulated in the experiment. An uninterruptible power supply (UPS) maintains the power supply to loads when public power is off. The UPS offers power with a base frequency of (50%±0.5%) Hz and voltage in (220%±3%) V. When switching the power from public power to UPS, a frequency variance occurs. The frequency fluctuation is shown in Figure 13, and the base frequency is calculated by Fourier transfer. From 0–30 s, the base frequency fluctuates around 49.99–50.02 Hz. At time point =30 s, the power supply swiftly changes to UPS, and base frequency is steady at 49.99 Hz, which fulfills the requirement of power supply. The power supply switch leads to frequency fluctuations of 0.01 Hz. Harmonic identification is examined in this section to test the effects of various OOSM algorithms on harmonic analysis. Harmonic source identification is one of the key issues in harmonic analysis and a necessity of smart grid safety. Harmonic identification analyzes multi-channel electrical data in a period and is affected by out-of-sequence measured data. A widely-applied method for harmonic identification is independent component analysis (ICA); its principle and realization have been explained in [41]. In this paper, this method is utilized to testify to the effects of disordered sequences on harmonic source identification accuracy.

**Figure 14:**
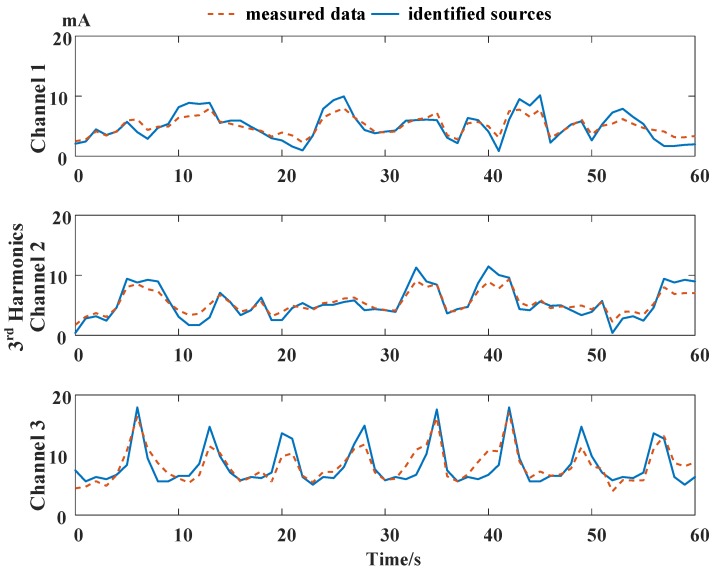
Third current harmonics of 3 typical channels at the same PCC with base frequency changes.

The harmonic series are measured under the distributed power sensing network the same as in Cases I and II. The limits of data disorders do not exceed 4 lags. Electrical parameters in 100 channels are collected. The harmonic sources are detected by Fluke 435 and compared to the measurement results of the sensing network. Three channels of the original electrical harmonic current measurement and the harmonic sources are shown in Figure 14. The 3 channels are linked to the same point of common coupling (PCC), and their harmonics affect each other. Harmonic source identification will separate the sources according to the independent component analysis method introduced in [41]. The 3rd–9th odd harmonics are measured to examine the effects of the OOSM algorithm on harmonic identification. The sampled electrical data and the correspondent identified harmonic sources’ profile are shown in Figure 14. The identification errors with 4 different OOSM methods are listed in Table 5.

The results indicate that the out-of-sequence phenomenon does have an influence on the precision of harmonic identification, and OOSM methods can help to decrease the error. The harmonic identification error may vary according to the identification method. However, with ICA, the NARX-based method gives the largest reduction in identification error among the 4 retrodiction methods. For the 7th and 9th harmonic, since the harmonic magnitudes are small, the harmonic identification error is unstable, the OOSM methods do not make much contribution to improving the precision. The data-aware method’s improvement on harmonic identification is verified.

### 4.7. Discussion

The experiment results prove the effectiveness of the retrodiction method on improving harmonic measurement accuracy in the cyber-physical energy system testbed. Considering that the out-of-sequence phenomenon is more general in a bandwidth-limited measurement network environment, the retrodiction method is more important. In the case of harmonic sensing, following the requirements in IEC 61000-4-7-2002, only SEPF and NARX-based methods fulfill the standard. This is mainly because the Kalman filter-based methods depend on a linear transmission matrix. The changes of the time-varying harmonics are not linear dynamics, but rather a non-Gaussian statistical model. The particle filter and neural network approximate the model with a Monte Carlo method, offering a better estimation accuracy. With a more complex structure, a neural network can approximate a more sophisticated model. Nevertheless, the improvement of the harmonic measurement precision over SEPF is not very manifest. This indicates that harmonic events can be depicted with a probabilistic model.

The computation cost of the NARX-based method stays stable within the maximum delay of harmonic measurements. This is because of the static structure of neural networks. Although it maintains a better real-time performance over other methods, it limits the application areas. In the harmonic measurement problem, the communication intervals are usually among several seconds, and transmission delay can be limited in a reasonable interval. When designing the NARX-based algorithm, the interval of the practical measurement network should be considered to define the maximum limit of the out-of-sequence delay. In other applications of the cyber-physical energy system, where a high real-time interaction and response with frequent communication among the network are required, the delay limit of out-of-sequence phenomenon can be large. If the size of the NARX-based method increases, the computation and memory cost might grow to a considerable scale. There should be further consideration when dealing with a larger network scale or complicated power quality analysis tasks.

In the harmonic measurement experiment, the results have demonstrated that the out-of-sequence measurement problem does exist, and the OOSM algorithm is capable of improving the harmonic analysis accuracy. In contrast, the measurement of the transient harmonic injection is more vulnerable to the OOSM problem than non-stationary harmonics; the retrodiction method also offers less reduction on the MSE of the transient harmonic measurement. This is because the transient harmonic injection is an independent event and cannot be well estimated by the kinematical function. For the non-stationary harmonic, its amplitude can be predicted to a certain extent; thus, the retrodiction accuracy is guaranteed. Thus, the modeling of the kinematical function is vital to ensure the harmonic measurement accuracy.

The OOSM algorithms use the Kalman filter, the particle filter and the neural network, which correspond to the linear kinematical function, Monte Carlo modeling and multi-layer modeling, respectively. With the complexity of the system arising, the modeling method also needs to be improved to approximate the physical behavior. In cyber-physical energy systems with a large-scale monitoring network, the events of harmonic changes are triggered by customer behaviors of a macro temporal probability distribution. However, in a particular unit, the dynamics of the harmonic cannot be approximated with the Monte Carol method or the Markov chain. From the experiment results, it can be figured that the neural network approximates the harmonic changes better than the Monte Carlo model, rather than the linear model. Thus, the neural network-based retrodiction method offers a better harmonic measurement and analysis accuracy. With a time-varying character, the NARX-based model can update the approximation model from the historical data. Thus, the model is updated by the data through time. Apparently, the harmonic measurement precision can be improved with better modeling of cyber-physical energy systems.

In a real dynamic power network, the system topology and the load types keep changing. This means that the dynamic function described in Equation (3) is not stable. If the NARX-based OOSM method still fulfills the requirements of online harmonic analysis, the time consumption of the neural network convergence to a new featured model should be limited. However, as the neural network will not be stable after decades or hundreds of training iterations, the time consumption is too long for the dynamic task. This problem can be solved by the idea of transfer learning. The NARX neural network is pre-trained under several load types: linear, stationary, transient, etc. The topology of the grid and the load type are identified continuously. If the input load type or the topology changes for the NARX neural network, the pre-trained weights are deployed. This shall reduce the time consumption to adjust to the new type of load. This is beyond the discussion of the OOSM method and is part of the future work.

Above all, the results suggest that the out-of-sequence measurement problem exists in large-scale distributed sensing networks, and the OOSM algorithm proposed can decrease the influence of the data disorders, which is helpful in online harmonic identification on cyber-physical energy systems.

## 5. Conclusions

In this paper, the out-of-sequence measurement problem is analyzed for asynchronous harmonic measurement in cyber-physical energy systems, and a new retrodiction method is proposed to improve the harmonic measurement accuracy. The proposed out-of-sequence measurement algorithm exploits the NARX neural network to approximate the dynamics of harmonic in distributed sensing networks and retrodicts the disordered datasets to improve the harmonic measurement precision. The performance of the NARX-based OOSM method is theoretically analyzed and compared to other retrodiction methods. The experiments examine the validation of the NARX-based OOSM algorithm. Results demonstrate that the NARX neural network can amend the electrical parameter data disorders while maintaining a relatively reasonable computation. Then, the harmonic measurement experiments with non-stationary and transient harmonics prove that the proposed OOSM algorithm can improve the harmonic measurement precision. Finally, the harmonic identification application is applied and validates the effectiveness of the retrodiction schedule on harmonic analysis.

In the future, the data-aware OOSM method will be examined under a hybrid and unsynchronized network and various transient electrical parameters. Theoretical analysis will be carried out on promoting the harmonic identification accuracy and real-time behavior in cyber-physical energy systems.

## Figures and Tables

**Table 1:** Computation complexity levels of OOSM algorithms: $n_{x}$ is the element number; *l* is the delay number; the maximum permitted delay is *L*; *m* is the number of measurements; $N_{w}$ is the number of total weights in the nonlinear autoregressive model with exogenous inputs (NARX) neural network; $p_{i}$ is the number of elements in each layer.

Algorithm	Computation Complexity Level
Bl	$ln_{x}^{2}+m\left(n_{x}^{3}+n_{z}^{3}\right)$
Lanzkron	$ln_{x}^{2}+m\left(n_{x}^{3}+n_{z}^{3}\right)$
ALG-S	$L^{3}n_{x}^{3}+L^{2}mn_{x}^{2}n_{z}+Lm^{2}n_{x}n_{z}^{2}+m^{3}n_{z}^{2}$
ALG-I	$L^{2}\left(n_{x}^{3}+n_{x}^{2}\right)+L\left(n_{x}^{3}+n_{x}^{2}\right)+mn_{x}^{3}+n_{x}^{3}$
A-PF	$ln_{x}^{3}+n_{x}^{2}$
SERBPF	$lm+mn_{x}$
NARX	$lN_{w}\approx lhn_{x}^{2}$

**Table 2:** Memory consumption of OOSM algorithms: *W* is the recursive time number, and $n_{x}$ is the element number.

Algorithm	Memory Consumption
Bl	$W\left(n_{x}^{2}+n_{x}\right)+Wn_{x}$
Lanzkron	$W\left(n_{x}^{2}+n_{x}\right)+Wn_{x}$
ALG-S	$Wn_{x}+W^{2}n_{x}$
ALG-I	$W\left(3n_{x}^{2}+2n_{x}\right)+Wn_{x}$
A-PF	$W(n_{x}^{3}+n_{x}^{2})$
SERBPF	$W(m+mn_{x})$
NARX	$W\left(m+l\right)\left(n_{x}+1\right)+Whn_{x}^{2}+Whn_{x}$

**Table 3:** Mean square errors of the non-stationary harmonic measurement accuracy (%).

Harmonics Order	Base		3rd		5th		7th		9th	
	Mean	Std	Mean	Std	Mean	Std	Mean	Std	Mean	Std
without OOSM	2.57	1.66	2.40	2.43	2.12	1.02	3.40	2.08	1.45	1.63
Bl	1.50	1.74	2.76	1.88	1.21	1.22	1.02	1.66	1.00	1.42
ALG-I	1.35	1.42	1.60	1.34	1.02	1.34	0.92	1.33	0.92	1.21
SEPF	1.25	1.32	1.50	1.24	0.90	1.08	0.90	1.12	0.87	0.97
NARX	1.02	1.21	1.33	0.95	0.92	1.23	0.90	1.01	0.88	0.95

**Table 4:** Mean square errors of the transient harmonic measurement accuracy (%).

Harmonics Order	Base		3rd		5th		7th		9th	
	Mean	Std	Mean	Std	Mean	Std	Mean	Std	Mean	Std
Bl	0.82	0.93	1.54	1.09	0.76	0.74	0.60	0.99	0.61	0.95
ALG-I	0.73	0.79	1.00	0.82	0.58	0.78	0.57	0.68	0.54	0.72
SEPF	0.65	0.71	0.91	0.63	0.59	0.59	0.55	0.61	0.47	0.55
NARX	0.55	0.62	0.87	0.60	0.61	0.65	0.57	0.62	0.55	0.59

**Table 5:** Mean square errors of the harmonic identification accuracy (%) with frequency variance.

Harmonics Order	Base		3rd		5th		7th		9th	
	Mean	Std	Mean	Std	Mean	Std	Mean	Std	Mean	Std
Bl	10.75	11.38	15.92	12.57	7.33	10.39	6.20	11.17	5.33	8.55
ALG-I	8.27	10.64	9.36	7.85	7.41	8.51	7.33	9.36	5.55	8.77
SEPF	7.27	7.54	9.82	8.76	7.28	6.70	5.99	9.13	5.96	7.24
NARX	5.57	8.70	7.45	6.83	7.37	8.59	6.09	7.49	5.59	6.88

## Abbreviations

The following abbreviations are used in this manuscript:

OOSM	Out-of-sequence measurement
CPES	Cyber-physical energy systems
AMI	Advanced infrastructure
NARX	Nonlinear autoregressive model with exogenous inputs
